# Biomarkers in Primary Focal Segmental Glomerulosclerosis in Optimal Diagnostic-Therapeutic Strategy

**DOI:** 10.3390/jcm11123292

**Published:** 2022-06-08

**Authors:** Aleksandra Musiała, Piotr Donizy, Hanna Augustyniak-Bartosik, Katarzyna Jakuszko, Mirosław Banasik, Katarzyna Kościelska-Kasprzak, Magdalena Krajewska, Dorota Kamińska

**Affiliations:** 1 Department of Nephrology and Transplantation Medicine, Wroclaw Medical University, 50-556 Wroclaw, Poland; hanna.augustyniak-bartosik@umw.edu.pl (H.A.-B.); katarzyna.jakuszko@umw.edu.pl (K.J.); miroslaw.banasik@umw.edu.pl (M.B.); katarzyna.koscielska-kasprzak@umw.edu.pl (K.K.-K.); magdalena.krajewska@umw.edu.pl (M.K.); dorota.kaminska@umw.edu.pl (D.K.); 2 Department of Clinical and Experimental Pathology, Division of Clinical Pathology, Wroclaw Medical University, 50-556 Wroclaw, Poland; piotr.donizy@umw.edu.pl

**Keywords:** primary FSGS, biomarkers, glomerulonephritis

## Abstract

Focal segmental glomerulosclerosis (FSGS) involves podocyte injury. In patients with nephrotic syndrome, progression to end-stage renal disease often occurs over the course of 5 to 10 years. The diagnosis is based on a renal biopsy. It is presumed that primary FSGS is caused by an unknown plasma factor that might be responsible for the recurrence of FSGS after kidney transplantation. The nature of circulating permeability factors is not explained and particular biological molecules responsible for inducing FSGS are still unknown. Several substances have been proposed as potential circulating factors such as soluble urokinase-type plasminogen activator receptor (suPAR) and cardiolipin-like-cytokine 1 (CLC-1). Many studies have also attempted to establish which molecules are related to podocyte injury in the pathogenesis of FSGS such as plasminogen activator inhibitor type-1 (PAI-1), angiotensin II type 1 receptors (AT1R), dystroglycan(DG), microRNAs, metalloproteinases (MMPs), forkheadbox P3 (FOXP3), and poly-ADP-ribose polymerase-1 (PARP1). Some biomarkers have also been studied in the context of kidney tissue damage progression: transforming growth factor-beta (TGF-β), human neutrophil gelatinase-associated lipocalin (NGAL), malondialdehyde (MDA), and others. This paper describes molecules that could potentially be considered as circulating factors causing primary FSGS.

## 1. Introduction

Focal segmental glomerulosclerosis (FSGS) is a pattern of glomerular lesion resulting from podocyte injury characterized by the occlusion of glomerular capillary loops by sclerotic material [1]. Sclerosis is focal (less than 50% of all glomeruli affected with light microscopy) and segmental (less than 50% of the glomerular tuft affected) [2]. It is the most common cause of nephrotic syndrome in adults in the USA and it recurs in 30–40% of patients after renal transplantation [3]. In the Polish adult population in a nationwide study, FSGS was the second most common diagnosis in renal biopsies (15% of cases, 16.2–16.8% of nephrotic syndrome) [4]. In the Italian population, FSGS accounted for 12.3% of nephrotic syndrome, which was similar to other European studies [5,6,7].

Clinical manifestations of primary FSGS are massive edemas, rapid onset of heavy nephrotic-range proteinuria, and severe hypoalbuminemia [8].

The diagnosis is established based on renal biopsy. FSGS classification is based on morphologic changes in the Columbia classification published in 2004, which distinguishes five variants: collapsing, tip, cellular, perihilar, and not otherwise specified (NOS) variant [1]. The Columbia classification is presented in Table 1.

The KDIGO (Kidney Disease: Improving Global Outcomes) guidelines proposed another classification that distinguished four types of FSGS: primary, secondary, genetic, and FSGS of undetermined cause [9], which are presented in Table 2.

Primary FSGS is a clinical-pathologic syndrome supposed to be caused by circulating permeability factors that lead to podocyte injury with podocyte foot process effacement. The high incidence of recurrence in the kidney allograft of proteinuria suggests that the circulating factor is present in recipients [10]. This hypothesis is confirmed by the fact that the implementation of plasmapheresis reduces proteinuria and decreases glomerular permeability. However, to date, post-transplant FSGS is the only form of FSGS that has been attributed to a circulating factor. The etiology of primary FSGS is still unidentified. It is known that antigens localized on podocytes may have a key role in the initiation and progression of the lesion observed in FSGS [11,12].

The causes of secondary FSGS include, but are not limited to, drug toxicity, adaptive changes to glomerular hyperfiltration, or virus-associated issues.

Genetic FSGS is typically present in early childhood and can be a familial, syndromic, or sporadic disease. Inheritance patterns are observed as autosomal dominant, autosomal recessive, X-linked, or mitochondrial (matrilineal) [13]. Genetic FSGS may either be limited to the kidney or be part of a broader syndrome with extrarenal involvement. It typically manifests in early childhood. The most commonly seen monogenic forms of FSGS are probably all known including the gene coding of slit diaphragm structure proteins, actin cytoskeleton, or cell signaling, but new causative genes continue to be identified with a growing number of variants of potential clinical significance [14] A study on a pediatric population with steroid resistant nephrotic syndrome/FSGS identified genetic variants of 37 known genes in 41% of patients including genes coding podocin (*NPHS2*), phospholipase C (*PLCE1*), RHO GTPase-activating protein 24 (*ARHGAP24*), Wilms tumor protein (*WT1*), and inverted formin 2 (*INF2*) [15]. Genetic FSGS caused by podocyte function abnormalities is usually resistant to corticosteroids. Calcineurin inhibitors may be more effective in therapy due to the stabilization of the podocyte actin cytoskeleton [13].

The nature of circulating permeability factors is not explained and particular biological molecules responsible for inducing FSGS are still unknown.

Several substances have been proposed as potential circulating factors such as soluble urokinase-type plasminogen activator receptor (suPAR) and cardiolipin-like-cytokine 1 (CLC-1). There have also been many studies that have attempted to establish which molecules are related to podocyte injury in the pathogenesis of FSGS such as plasminogen activator inhibitor type-1 (PAI-1), angiotensin II type 1 receptors (AT1R), dystroglycan (DG), microRNAs, metalloproteinases (MMPs), forkheadbox P3 (FOXP3), and poly-ADP-ribose polymerase-1 (PARP1). Some biomarkers have also been studied in the context of kidney tissue damage progression: transforming growth factor-beta (TGF-β), human neutrophil gelatinase-associated lipocalin (NGAL), malondialdehyde (MDA), and others [10].

This paper describes molecules that could potentially be considered as circulating factors causing primary FSGS. The biomarkers can be divided into three groups:

- Potential circulating permeability factors (Table 3),
- Biomarkers potentially contributed to podocyte injury in the pathogenesis of FSGS (Table 4);
- Biomarkers of kidney injury progression (Table 4).

We also present the biomarkers of possible influence on podocyte injury that have not been studied in FSGS thus far, but may be of interest for future research.

## 2. Potential Circulating Permeability Factors

### 2.1. Soluble Urokinase-Type Plasminogen Activator Receptor

Soluble urokinase-type plasminogen activator receptor (suPAR) is the soluble form of the urokinase-type plasminogen activator receptor (uPAR) and is a membrane-bound 45–55 kDa protein [77]. It expresses on many immunologically active cells (e.g., monocytes, neutrophils). SuPAR is a glycosylphosphatidylinositol (GPI)-anchored three-domain (DI, DII, and DIII) protein (such as β-integrins of leukocytes and b3 integrin in podocytes). It is responsible for signaling complexes with various transmembrane proteins including integrin, caveolin, and G-protein-coupled receptors [10]. Elevated levels of suPAR in plasma have been observed in immune system activation, in many inflammatory diseases (sepsis, malaria, tuberculosis, rheumatoid arthritis, HIV), malignant neoplasms (e.g., ovarian cancer), and also in FSGS [57,77,78].

In the experimental model of FSGS, it was shown that circulating suPAR activates the podocyte β3 integrin in both native and grafted kidneys, causing foot process effacement, proteinuria, and FSGS-like glomerulopathy [16]. Recent experimental study on human podocytes and two mouse models showed that suPAR by itself is not the cause for direct podocyte injury, *in vitro* or *in vivo* [17].

Some experimental studies have been published on the relation of suPAR to proteinuria therapy. Zhang et al. found that amiloride, a potassium-sparing diuretic, could inhibit uPAR expression and the activation of the β3 integrin of podocytes *in vitro* and also in 5/6 nephrectomy rat models *in vivo*. This experimental study showed that amiloride could reduce proteinuria and glomerular sclerosis [79].

Wei et al. observed that significantly elevated suPAR serum concentrations were reported in patients with FSGS when compared to healthy subjects [16]. Significantly increased suPAR serum levels in FSGS and lower in patients with membranous nephropathy or minimal change disease and lowest in normal individuals were also reported. There was no significant difference in the suPAR levels between the patients with primary and patients with secondary FSGS. The suPAR levels increased in the order of tip variant, to a not otherwise specified variant and a cellular variant [20].

Sun et al. compared the suPAR concentrations in the most common types of secondary FSGS (Alport-FSGS, obesity-related FSGS, diabetic nephropathy-related FSGS) to a control group (56 healthy donors and 74 patients with primary FSGS, 14 with MCD, and 29 with membranous nephropathy). In the study group, the plasma suPAR levels were higher compared to the control group. The authors also showed that the plasma suPAR level in the patients with secondary FSGS was higher than in the primary FSGS. On the other hand, the urinary suPAR level was lower in the secondary than in the primary FSGS. They did not find a correlation between the increased suPAR and decreased GFR, however, this study had a limitation in the number of patients (only 52 cases) [21]. A recent study showed that patients with treatment-resistant primary FSGS with a high suPAR level and evidence of podocyte activation were resistant to rituximab therapy [22]. The study by Winnicki et al. showed that patients with FSGS presented significantly higher suPAR levels than patients with other glomerulonephritis and healthy individuals. This applied to subjects with and without chronic kidney disease. Moreover, a higher plasma suPAR level was predictive for progression to end-stage renal disease [23].

A more recent study by Sun showed that both the plasma and urinary suPAR levels in the secondary FSGS were significantly higher than in the healthy controls. There was no significant difference in the levels of plasma and urinary suPAR in the Alport-FSGS, obesity-related FSGS, and diabetic nephropathy groups. Moreover, the plasma suPAR levels were not correlated with the kidney filtration function and proteinuria [24].

Many studies have shown that higher suPAR level correlated with a decreased level in eGFR [8,18,25,26].

Elevated serum suPAR was observed in all transplant candidates with advanced renal disease compared with the healthy controls, however, it did not differentiate primary kidney disease diagnosis. Pretransplant urine but not serum suPAR levels were reported to identify cases of recurrent FSGS in kidney transplant candidates [27].

SuPAR was postulated to be a predictive marker of kidney allograft outcome. Elevated plasma levels of suPAR were seen in recipients with recurrent FSGS after kidney transplantation, and the suPAR concentrations correlated with the presence but not with the degree of proteinuria. The SuPAR concentration of one year after kidney transplantation was higher in patients with recurrent FSGS compared to patients with normal kidney function. The incidence of recurrence of FSGS after transplantation was higher in patients with higher plasma suPAR levels before transplantation [16]. In contrast, a recent study showed that intact suPAR levels were not significantly different between the recurrent FSGS and non-recurrent FSGS after transplantation [19].

The above-mentioned studies showed that plasma suPAR could be a useful marker of glomerular diseases with various podocytopathies and an important pathogenetic factor of FSGS, but do not discriminate primary FSGS from secondary forms and thus cannot be regarded as the only circulating permeability factor.

### 2.2. Cardiolipin-Like-Cytokine 1

Cardiolipin-like-cytokine 1(CLC 1) is also known as cardiolipin-like-cytokine factor 1(CLCF 1), neurotrophin-1, or B-cell-stimulating factor 3 (NNT-1 or BSF-3) [24]. It belongs to the IL-6 family [24,58,80,81]. It takes part in the development of the central nervous system through the formation of motor neurons. CLC-1s transcripts are present in the skeletal muscle and in serum during massive motor neuron cell death [82,83].

It is postulated that CLC-1 regulates the activity of neutrophils and has immunomodulatory properties after its interaction with soluble receptor CLF (soluble receptor cytokine-like factor-1). This connection influences the proper formation of the extracellular matrix and its dysregulation leads to a disturbance in the concentration of collagen type I and III and the deposition in the liver, thereby finally causing fibrosis [84].

CLC-1 was postulated as a permeability factor in recurrent FSGS. The correlation between the decreased expression of nephrin (which influences the reorganization of actin cultured podocytes) and increased CLC-1 that altered glomerular permeability was described. Moreover, the monoclonal antibody against CLC-1 blocked the effect of FSGS sera on albumin permeability [80,85]. Other studies have suggested that the downregulation of JAK2/STAT3 signaling could relate to the circulating permeability factor by inhibiting CLC-1 [28]. The CLC-1 affinity for galactose could be blocked by galactose itself [85]. The use of galactose for the reduction in albumin permeability and proteinuria was investigated. However, the studies involved few individual patients with FSGS and still remain controversial. In 2015, Trachtman et al. published the results of the FONT Trial. They examined alternative treatments in FSGS such as adalimumab or galactose compared to the standard medical therapy. This trial showed that galactose therapy for 26 weeks could have a potential value in FSGS treatment, causing a reduction in proteinuria in 42% of the treated patients. However, more research studies with a higher number of patients with earlier stages of FSGS are necessary in order to establish the relationship between albuminuria and fibrosis [86,87,88].

In summary, the precise role of CLC-1 in the pathogenesis of primary as well as secondary FSGS is still unknown.

## 3. Biomarkers Potentially Contributed to Podocyte Injury or Cell Signaling in the Pathogenesis of FSGS

In the following paragraphs, we discuss the biomarkers that potentially contribute to podocyte injury or cell signaling, which may play a role in the pathogenesis of FSGS. We chose biomarkers that have been studied in primary FSGS or other types of glomerulopathies, especially MCD (angiotensin II type 1 receptor, metalloproteinases, microRNAs, dystroglycans). We also included biomarkers that have not yet been studied on FSGS, but may be of research interest: plasminogen activator inhibitor type, forkheadbox P3, and poly ADP-ribose polymerase-1.

The biomarkers are below sorted by decreasing confirmation level of the research data.

### 3.1. Angiotensin II Type 1 Receptor

Angiotensin II type 1 receptors (AT1R) are present on endothelial cells and podocytes. It is known that angiotensin-converting enzyme inhibitors and AT1R antagonists have a protective role in renal tissue damage and proteinuria.

It is supposed that angiotensin II type 1 receptor antibodies (AT1R-Abs) dysregulate AT1R receptors, which leads to podocyte injury, glomerular endotheliosis, and subsequent proteinuria [29]. In animals, AT1R-Abs have been shown to induce malignant hypertension, preeclampsia, and post-transplant FSGS [30]. In an experimental model of glomerulonephritis, AT1R activation hampered mRNA expression of the molecules of the slit diaphragm, which led to proteinuria [89].

AT1R-Abs is associated with vascular rejection of kidney allografts in the absence of human leukocyte antigen antibodies (anti-HLA) [90]. A case report of AT1R-Abs present with newly diagnosed collapsing FSGS and antibody-mediated rejection 1 month after renal transplantation was published [91]. It was also observed that the expression of AT1R in the tubular epithelium of biopsy samples was associated with a significantly higher risk of graft loss [31]. Moreover, the presence of AT1R-Abs was described as a risk factor for transplant injury. AT1R expression in renal transplant biopsy may help predict transplant rejection. Both anti-AT1R antibodies in serum and the expression of AT1R in kidney allograft biopsies could be useful biomarkers in the prediction of graft loss [32].

The role of AT1R-Abs in the pathogenesis of FSGS recurrence after renal transplantation was evaluated. Correlation between AT1R-Abs and post-transplant FSGS recurrence was observed. Increased serum levels of AT1R-Abs in patients with recurrence FSGS after transplantation were observed, suggesting that AT1R-Abs may be a useful marker for post-transplant FSGS [33,34].

The role of AT1R in primary FSGS has also been evaluated in some studies. Polymorphism of the renin-angiotensin system in children with nephritic syndrome was an important risk factor in the prognosis in progression to end-stage renal disease. The polymorphism is related to the insertion or deletion of the 287 bp fragment of intron 16 in the ACE gene. The other polymorphisms are A1166C polymorphism in AT1R and M235T polymorphism in angiotensinogen. Type D allele of the ACE gene was described as a risk factor for the progression to ESRD in patients with FSGS. However, the authors underlined that genetic polymorphism is one of many factors determining prognosis and the most important is steroid response [92]. Similar findings were described by Frishberg et al., where the D allele had a dominant and adverse influence on renal function in young patients with FSGS [35].

### 3.2. Metalloproteinases and Tissue Inhibitors of Metalloproteinases

Metalloproteinases (MMP) are multidomain enzymes and zinc-containing endopeptidases. They are responsible for remodeling extracellular matrix, fibrosis, and are present in several organs including the kidney [36,36]. Both MMP-2 and 9 were found in the serum of patients with chronic kidney disease and in mesangial cells. Their elevated expression was observed in podocytes during inflammation [38,39,40]. Increased concentration of MMP-9 was found in many types of renal injury such as FSGS, IgA nephropathy, nephritis in Henoch-Schonlein disease, and post-streptococcal glomerulonephritis [39,93,94]. MMP-9 also takes place in the pathology of glomeruli in HIV-associated nephropathy [95]. It is known that the MMP-9/NGAL ratio is a better biomarker in the differentiation of MCNS (minimal change nephrotic syndrome) and FSGS in children with nephrotic syndrome than the individual use of each molecule [94,96]. MMP-2 concentrations were increased in tubulointerstitial fibrosis in patients with diabetic nephropathy [97].

In renal transplant recipients, proteinuria was significantly associated with elevated concentrations of plasma MMP-2 and urine MMP-2. In plasma, increased tissue inhibitors of metalloproteinases were also observed, which inhibit the degradation of the extracellular matrix (ECM) by MMPs and lead to excessive deposition of ECM proteins [98]. Czech et al. compared the concentration of MMPs and their inhibitors in patients with FSGS to patients with steroid-sensitive nephrotic syndrome. The level of MMPs and tissue inhibitors of metalloproteinases (TIMPs) were higher in patients with FSGS [41]. In patients with FSGS, increased MMP-2 and unchanged MMP-9 plasma concentrations were observed [38]. MMP-2 was used to investigate the response to treatment in patients with FSGS. Patients were divided into two groups, steroid-sensitive and non-sensitive, depending on their response. In the results, MAPK/MMP-2 was observed to be upregulated in glomeruli isolated from steroid-non-sensitive patients [99].

### 3.3. Dystroglycans

Dystroglycans are components of the neuromuscular junction. Dystroglycan (DG) is an important element of the dystrophin–glycoprotein complex (DGC), which connects the subsarcolemmal cytoskeleton to the basal lamina in skeletal muscle [100].

The dystroglycan gene is expressed as a precursor protein that is posttranslationally divided inside into an extracellular peripheral membrane protein called alpha-dystroglycan (156 kDa) and a transmembrane protein, beta-dystroglycan (43 kDa) [42,43,100,101,102,103,104,105,106].

Mutations in genes encoding glycosyltransferases that cause glycosylation and extracellular matrix binding activity of a-dystroglycan (a-DG) prompt congenital muscular dystrophies (CMDs) with symptoms from the central nervous system [107].

In the kidney, alpha and beta DGs mechanically anchor the podocyte cytoskeleton to the glomerular basement membrane [100,108]. Beta-DG transports important matrix proteins such as perlecan, agrin, and proteoglycans [42,43].

Studies about the role of DG in glomerulopathies are ambiguous. A decreased concentration of alpha and beta dystroglycan has been described in patients with MCD in comparison with the normal level in cases with FSGS [44]. The expression of β-chain DG in the podocytes did not differ in MCD and FSGS and, moreover, the presence of β-chain DG in podocytes did not influence the steroid sensitivity [45]. On the other hand, Giannico et al. observed increased DG expression in renal biopsies in patients with FSGS [44,109].

### 3.4. MicroRNAs (miR-192, miR205, and miR-186)

MicroRNAs (miRNAs, miRs) are responsible for many physiological, developmental, and pathological processes. MicroRNAs are present in serum, plasma, and urine [110,111]. MiR-192 and miR-205 are highly organ-specific and expressed in the renal cortex, while other MicroRNAs are present in many tissues. MiR-192 and miR-205 were revealed to be more abundant in the kidneys than in other organs in rats. MicroRNAs seem to be perfect biomarkers because they are comparatively stable.

In transplantology, miRNAs take part in processes occurring after renal transplantation including delayed graft function (DGF) and acute rejection. Seven miRNAs were identified in DGF kidneys (miR-182, miR-106b, miR-20a, miR-21*, miR-18a, miR-17, and miR-106a). Mir-182-5p is described as a key regulator of postischemic acute kidney injury. In acute cellular rejection, four miRNAs (miR-150, miR-155, miR-663, miR-638) were identified as important upregulators [46].

There are 48 miRNAs associated with IgAN. Four of them, miR-148a-3p, miR-150-5p, miR-20a-5p, and miR-425-3p, had increased expression in patients with IgAN relative to the control group. Dozens of microRNAs were described as connected with lupus nephritis. Some were used as markers for early stages of the disease, while some were examined in renal failure and some in predicting proteinuria [112].

The serum concentration of miR-192 and miR205 was increased in FSGS cases versus patients with MCD [47]. Overexpression of miR-193a in mouse podocytes induced foot process effacement and a catastrophic collapse of the entire podocyte-stabilizing system and eventually glomerulosclerosis by the inhibition of WT1, which controls the differentiation and homeostasis of podocytes and maintains the expression of several genes that are essential for the podocyte structure. Moreover, the upregulation of miR-193a was observed in the glomeruli of FSGS patients [48,49].

Below, we present the biomarkers of possible influence on podocyte injury or cell signaling that have not been studied in FSGS thus far, but may be of interest for future research.

### 3.5. Plasminogen Activator Inhibitor Type-1

Plasminogen activator inhibitor type-1 (PAI-1) is involved in tissue homeostasis by inhibiting plasmin-mediated metalloproteinase (MMP) activation. Many studies have suggested that deficiency or inhibition of PAI-1 activity causes fibrosis in different organs (liver, lung, and kidney). The dysregulation of plasmin-mediated MMP activity attenuates collagen degradation and its increased accumulation. This process causes fibrotic matrix deposition in tissues [50].

Dysfunction of PAI-1 leads to fibrin accumulation in vessels, which can cause atherothrombosis. This process is especially observed in patients with type 2 diabetes mellitus. Increased expression of PAI-1 in vessels leads to vascular inflammation by promoting neointima formation and inhibiting the clearance of platelet fibrin thrombi. In oncology-related studies, elevated PAI-1 concentrations have been observed in various types of tumor tissues or plasma compared with the controls. Furthermore, PAI-1 is useful as a prognostic factor, particularly in breast cancer. It also promotes tumor vascularization and causes cell dissemination and the formation of metastases [113].

Hamano et al. investigated the concentration of PAI-1 in different primary and secondary glomerulopathies in 80 renal biopsy specimens. Glomerular PAI-1 mRNA expression was significantly higher in patients with MCD and FSGS in comparison to patients with other types of glomerulopathies. Moreover, PAI-1 mRNA expression correlated with the level of proteinuria, which suggests that the increased expression of PAI-1 mRNA in glomeruli could be an important factor in the progression of glomerular changes in various types of glomerulonephritis [51].

It was shown that fibrinolytic activity dependent on PAI-1 could promote the development of glomerulonephritis. Lee et al. analyzed the expression of PAI-1 on podocytes in IgA nephropathy and found a strong reactivity of PAI-1 in the proximal tubules. However, no correlation between the plasma concentrations of PAI-1 and its staining intensity or pattern of expression on the podocytes and tubules was observed. It was found that the serum PAI-1 levels did not correlate with the clinical parameters [52]. Another study described the increased expression of PAI-1 in the mesenchyme of glomerular and renal tubules with the increasing severity of the mesangial proliferative glomerulonephritis. This suggests that PAI-1 plays an important role in the development of mesangial proliferative glomerulonephritis [53].

By inhibiting plasminogen activators and reducing glomerular mesangial matrix turnover, PAI-1 decreases plasmin generation and plasmin-mediated matrix degradation. Therapy with a mutant human PAI-1 (PAI-1R), which connects to the vitronectin matrix but does not inhibit the plasminogen activators, could increase plasmin generation and decrease the matrix accumulation in experimental glomerulonephritis. PAI-1R reduced glomerulosclerosis due to competing with endogenous PAI-1, remodeled plasmin generation, and inhibited inflammatory cell infiltration. This led to a decreased local production of TGF-β1 and reduced matrix accumulation [54].

The particular role of the PAI-1 system in FSGS has not been elucidated thus far.

### 3.6. Forkhead Box P3

Forkheadbox P3 (FOXP3) is a transcription factor that influences the maintenance of the regulatory T-cell (Treg) phenotype and function. Tregs preserve immune homeostasis by the inhibition, activation, and influence on the function of other leukocytes. They are responsible for the suppression of inflammation. Mutation of the FOXP3 gene leads to immunodysregulation, polyendocrinopathy, enteropathy, X-linked (IPEX) syndrome with dermatitis, enteropathy, diabetes, thyroid disorders, and anemia [114].

The role of Tregs and FOXP3 in the pathogenesis of proliferative and crescentic forms of glomerulonephritis was evaluated. Experimental studies on the depletion of regulatory T-cell (DEREG) mice that express the diphtheria toxin receptor under control of the foxP3 (forkhead box P3) gene promotor were performed. Treg cell dysregulation caused glomerulonephritis with glomerular crescent formation [55].

Other studies on FOXP3 mRNA expression showed an important role in the long-term outcomes for kidney allografts and it was shown to be useful in the identification of recipients at high risk for acute rejection. In a quantitative analysis of FOXP3 mRNA in peripheral blood mononuclear cells (PBMCs) and urine-sediment cells of patients with acute rejection, Barabadi et al. revealed a correlation between the concentration of FOXP3 mRNA in urinary sediment and PBMCs. A negative correlation was observed between the levels of FOXP3 mRNA and serum creatinine concentration during an episode of acute rejection. The authors suggested that monitoring of FOXP3 mRNA in PBMCs and urine may be useful for predicting kidney allograft outcome [56].

In renal transplant biopsies, FOXP3 mRNA expression was increased in rejection (T-cell and antibody-mediated) compared to non-rejection samples and correlated with T-cell-associated transcripts, younger donor age, and longer time posttransplant. This indicated that the expression of FOXP3 mRNA is a time-dependent feature of inflammatory infiltrates in kidney tissue [115]. Expression of FOXP3 was elevated directly after kidney transplantation. On the other hand, in recipients with chronic rejection, lower levels of FOXP3 expression have been described [116].

Thus far, there have been no published studies on FOXP3 expression in the context of FSGS. However, its influence on the kidney allograft outcome suggests a possible role in FSGS and in other types of glomerulonephritis.

### 3.7. Poly ADP-Ribose Polymerase-1

Poly ADP-ribose polymerase-1 (PARP-1) is a nuclear protein that regulates gene expression as a coactivator of transcription and protein functions via poly(ADP ribosyl)ation. Poly(ADP ribosyl)ation has a multifaceted influence on protein activation as well as downregulation. The activation of the poly(ADP-ribose) polymerase (PARP) plays a role in the pathophysiology of various diseases associated with oxidative stress. PARP-1 repairs DNA damage and maintains genome integrity [117,118].

Many studies on PARP-1 as a multifunctional molecule with a therapeutic potential have been performed in various medical fields.

In oncology, it has been shown that the elevated expression of PARP-1 is an independent negative prognostic marker in mucosal melanomas [67]. Nuclear-cytoplasmic PARP-1 expression was also an unfavorable prognostic marker in lymph node-negative early breast cancer [119]. PARP-1 activation is responsible for cisplatin-induced cell death and tissue injury by oxidative stress. Mukhopadhyay et al. suggested that PARP-1 inhibition may prevent cisplatin-induced nephrotoxicity [120].

In neurology, the PARP-1 inhibitor has been described as adjunctive therapy for treatment in the early stage of Alzheimer’s disease [121]. In cardiology, pharmacological inhibition of PARP-1 may exert a beneficial influence in the alleviation of myocardial ischemia, different types of heart failure, cardiomyopathies, circulatory shock, cardiovascular aging, and cardiovascular complications connected with diabetes mellitus [122].

In kidney allografts, PARP-1 expression was described to positively correlate with serum creatinine levels at biopsy as well as with delayed graft function [123]. On the other hand, inactivation of PARP-1 is thought to be protective in acute kidney injury-induced interstitial fibrosis [124]. This was confirmed in an experimental study by Zhengs et al., who showed that gene ablation of PARP-1 protects the kidneys from ischemic injury [125].

An important role of PARP-1 in chronic renal dysfunction was reported by Słomińska et al., who observed elevated levels of nicotinamides Met2PY and Met4PY in the plasma of children with chronic renal disease. High levels of Met2PY and Met4PY inhibit PARP-1 activity. This mechanism was proposed to exert a beneficial influence and protect renal cells against oxidative stress injury, leading to cell apoptosis. In contrast, a correlation between the concentration of nicotinamide and renal insufficiency was observed. It was hypothesized that nicotinamide metabolites may be protective in the short-term whereas chronic exposure is probably detrimental because of the impairment of the DNA repair mechanism [126].

There have been no published studies on the direct correlation between PARP-1 and inducing FSGS. Nonetheless, PARP-1 is a molecule that influences detrimental processes related to oxidative stress. The foregoing research suggests that it could be an important mechanism initiating renal failure, leading to FSGS.

## 4. Biomarkers of Kidney Injury Progression

Below, we present the biomarkers of non-specific kidney injury progression that have been studied in FSGS, or have not been studied so far, but may be of interest for future research.

### 4.1. Transforming Growth Factor-Beta

Transforming growth factor-beta (TGF-β) is a cytokine with pleiotropic effects. It is responsible for differentiation, proliferation, and other immune functions in many types of cells. Numerous cells produce TGF-β and express its receptor. TGF-β controls many other growth factors positively and negatively [58,59,60,61,62].

Proliferative and fibrotic lesions in glomeluropathies are associated with elevated expression of tissue growth factors such as fibroblast growth factor (FGF), platelet-derived growth factor (PDGF), epidermal growth factor (EGF), and TGF-β. Integrin-linked kinase (ILK) is a protein involved in the pathogenesis of many nephropathies with proteinuria. An increased concentration of TGF-β induces ILK expression [127].

Pretransplant peripheral blood TGF-β gene expression was found to negatively influence immediate post-transplant kidney allograft function [128]. Compared with normal kidney tissue, a high gene expression of TGF-β1 was identified in kidneys retrieved from brain dead donors. Moreover, a higher decrease in TGF-β gene expression during warm ischemia was seen in kidney allografts with better function up to 24 months [129].

Increased TGF-β urinary concentration in patients with FSGS and IgAN compared to other types of glomerulopathy and healthy controls have also been reported. However, there was a significant correlation between the urinary TGF-beta levels and the grade of interstitial fibrosis [130]. Glomerular diseases characterized by extracellular matrix accumulation (including FSGS) all showed a significantly increased expression of the three TGF-beta isoforms in glomeruli and the tubulointerstitium [63]. TGF-β promotes podocyte apoptosis, proliferation, and matrix deposition [64,65]. It also causes hypertrophy of the glomeruli, leading to high intracapillary pressure and protein loss [77,131,132]. TGF-β synthesis is controlled by cell-cycle regulatory proteins positively and negatively. An increased TGF-β1 gene expression was observed in biopsies in patients with progressive FSGS. TGF-β1 gene expression was noted in 18 of 20 steroid-resistant (SR) children with nephrotic syndrome caused by FSGS compared to 3 of 14 steroid-sensitive patients. Later, two of these patients developed FSGS. This suggests that the transcription of the TGF-β1 gene could be a predictive marker of renal failure in FSGS [66]. Experimental studies showed that TGF-β mRNA in the kidneys was absent in the early-phase nephrotic syndrome but could later induce the accumulation of extracellular matrix. Elevated urinary TGF-β levels were observed in FSGS cases in the late stage of the disease, where sclerotic lesions were advanced [133].

Smad2, Smad3, and Smad7 are molecules that are stimulated by TGF-β receptors. Elevated expression of their phosphorylated (active) forms was observed in patients with FSGS [134,135,136]. Smad7 was found to play a role in podocyte injury by inducing cell apoptosis [136]. On the other hand, Smad7 could inhibit active forms of Smad2 and Smad3 by stopping their phosphorylation [135].

The results of many studies suggest that TGF-β could be an ideal candidate for a biomarker, which is observed in kidney lesions in FSGS [137]. However, its precise role in the pathogenesis of FSGS has not been elucidated yet.

### 4.2. Human Neutrophil Gelatinase-Associated Lipocalin

Human neutrophil gelatinase-associated lipocalin (NGAL), also referred to as Lipocalin-2, Siderocalin, Uterocalin, and 24p3, is a 25 kDa protein covalently connected to gelatinase in human neutrophils. It is found in several human tissues including the kidney, trachea, lungs, stomach, and colon at very low concentrations [68].

Increased serum levels of NGAL in patients with renal dysfunction have been shown [69]. NGAL is a renal lipocalin that is highly accumulated in the human proximal tubule during ATN and its synthesis is significantly upregulated within hours of ischemia-reperfusion injury. Urine NGAL is an early predictor of AKI. This increase in NGAL precedes the elevation in serum creatinine by several hours to days [68,69,70].

In pathology, isoenzymes of NGAL are observed in glomeruli and renal tubular cells, which causes elevated urinary excretion of NGAL, while at the same time, filtration is not elevated because of the injury in the glomerular capillary wall [71,138]. Zhang et al. confirmed that urinary NGAL is a good biomarker of tubulointerstitial lesions in FSGS patients and could be useful in diagnosing FSGS, detecting acute tubulointerstitial lesions, and predicting treatment response [47]. Urinary NGAL in children with FSGS was used as a marker of chronic kidney disease progression with a decrease in both the glomerular filtration rate and the level of proteinuria [138].

### 4.3. Malondialdehyde

Malondialdehyde (MDA) is the main end-product of the degradation and autoxidation of polyunsaturated fatty acids or their esters formed in all cells [72]. MDA is genotoxic and cytotoxic and it takes part in the pathogenesis of different diseases and pathologic processes (diabetic nephropathy, aging and Alzheimer’s disease, radiation damage, mutagenesis, and carcinoma). MDA is a biomarker of oxidative stress. Elevated MDA urinary levels induced by oxidative stress and causing lipid peroxidation were observed in patients with chronic kidney disease [73,74,75,79,139,140].

Kuo et al. observed elevated levels of MDA in plasma, urine, and glomeruli in patients with FSGS with normal kidney function [76]. They also found that the increased concentration of MDA in urine significantly impacted the sclerosing score of glomerulosclerosis. In contrast, in patients with MCD, the urinary MDA level was not increased. This fact once more indicates that oxidative stress takes place in the pathogenesis of FSGS. Moreover, the glomerular MDA level in kidney biopsies correlated well with the degree of glomerulosclerosis in patients with idiopathic FSGS [76].

Further studies are needed on the role of MDA in the development of FSGS as well as on the prognostic value of MDA in this disease.

## 5. Conclusions

A better understanding of the pathogenesis of FSGS and the role of various molecules in the pathology of podocyte injury may lead to the implementation of etiology-targeted therapy. The above-mentioned biomarkers are proposed to be involved in decreasing the permeability of the glomerular membrane. The multitrophic mechanism of action of the presented biomarkers (immunomodulation, modification of gene expression, in oxidative stress process or in the cell matrix) showed that podocyte injury is a result of heterogeneous processes. Further studies are needed to discover the permeability factor or factors and to translate the experimental data into clinical settings.

## Figures and Tables

**Table 1:** The Columbia classification of FSGS.

FSGS/Variant	Inclusion Criteria	Exclusion Criteria
**FSGS not otherwise specified (NOS)**	At least 1 glomerulus with segmental increase in matrix obliterating the capillary lumina. There may be segmental glomerulus capillary wall collapse without overlying podocyte hyperplasia.	Exclude perihilar, cellular, tip, and collapsing variants.
**Perihilar variant**	At least 1 glomerulus with perihilar hyalinosis, with or without sclerosis >50% of glomeruli with segmental lesions must have perihilar sclerosis and/or hyalinosis.	Exclude cellular, tip, and collapsing variants
**Cellular variant**	At least 1 glomerulus with segmental endocapillary hypercellularity occluding lumina, with or without foam cells and karyorrhexis.	Exclude tip and collapsing variants.
**Tip variant**	At least 1 segmental lesion involving the tip domain (outer 25% of tuft next to origin of proximal tubule). The tubular pole must be identified in the defining lesion. The lesion must have either an adhesion or confluence of podocytes with parietal or tubular cells at the tubular lumen or neck. The tip lesion may be cellular or sclerosing.	Exclude collapsing variant. Exclude any perihilar sclerosis
**Collapsing variant**	At least 1 glomerulus with segmental or global collapse and overlying podocyte hypertrophy and hyperplasia	None

**Table 2:** The KDIGO classification of FSGS.

Etiology			
**Primary**	FSGS with diffuse process effacement and nephritic syndrome (often sudden onset amenable to therapy		
**Secondary**	Adaptive changes to glomerular hyperfiltration (normal or reduced nephron mass, segmenta foot process effacement, proteinuria without nephritic syndrome	Viral: HIV, probably: HCV, CMV, parvovirusB19	Drug induced: mTOR inhibitors, calcineurin inhibitors, anthracyclines, heroin(adulterants), direct-acting antiviral therapy (ledipasvir, sofosbuvir, heroin (adulterants), lithium, IFN, anabolic steroids
**Genetic**	Familial	Syndromic	Sporadic
**FSGS of undetermined cause**	Segmental foot process effacement	Proteinuria without nephritic syndrome	No evidence of secondary cause

**Table 3:** The circulating permeability factors.

Biomarkers	Analyzed Variables	Population	Results	Clinical Utility	References
**suPAR (experimental)**	The effect of suPAR on activation of podocyte β3 integrin expression in native and graft kidneys	Three mice models	Renal damage develops when suPAR activates β3 integrin	Not known	[16]
	Proteinuria/kidney pathology	A primary culture of human podocytes and two mouse models	Amiloride inhibits podocyte uPAR induction and reduces proteinuria	Identification of amiloride anti-proteinuric properties	[17]
	Proteinuria	Mouse model	Injection of recombinant suPAR in wild-type mice did not induce proteinuria within 24 h	suPAR do not induce proteinuria	[18]
**suPAR** **(clinical)**	suPAR serum level (ELISA)	78 pts with FSGS, 25 pts with MCD, 7 pts with preeclampsia, 16 pts with MN, 22 healthy subject	Positive correlation between increased suPAR and decreased eGFR; suPAR elevated in 2/3 of pts with primary FSGS but not in other glomerular diseases	Identification serum suPAR as a circulating factor	[16]
	Circulating levels of individual soluble suPAR forms to assess the risk of FSGS recurrence after transplantation (ELISA, TR-FIA)	55 pts with primary FSGS, 15 pts with non-FSGS glomerular diseases, 15 healthy subjects	- suPAR levels significantly elevated in FSGS compared to healthy controls; - large variability in suPAR levels in FSGS with not significantly higher suPAR levels compared to glomerular diseases - suPAR levels did not differ between recurrent and non-recurrent FSGS after KTx	- suPAR using the commercial ELISA not appear to be a useful marker for FSGS - suPAR(I) assessed by TR-FIA assay is a potential biomarker for FSGS	[19]
	Plasma suPAR level (ELISA)	74 pts with primary FSGS 14 pts with secondary FSGS controls: healthy subjects, MCD, MN	- suPAR levels in primary FSGS significantly higher compared to MCD - suPAR levels negatively correlated with creatinine clearance at presentation but positively correlated with crescent formation in primary FSGS	suPAR do not differentiate primary and secondary FSGS	[20]
	Serum suPAR level (ELISA)	164 pts with primary FSGS (the North America–based FSGS clinical trial and PodoNet)	- suPAR levels elevated in the majority of primary FSGS - an negative correlation of suPAR to eGFR	suPAR levels correlate with remission	[21]
	Plasma suPAR level (ELISA)	38 pts with primary FSGS	Rituximab was ineffective at producing a sustained remission	Sustained high suPAR levels are marker of disease resistance to treatment	[22]
	Plasma suPAR level (ELISA)	7 pts with primary FSGS, 21 pts with secondary FSGS 6 pts with recurrence of FSGS after KTx, 32 healthy subjects, 60 pts with GN	- Higher suPAR levels in FSGS; - suPAR levels do not differ between primary and FSGS or recurrent FSGS after KTx;	Higher suPAR predictive for progression to ESRD	[23]
	Plasma and urinary suPAR levels (ELISA)	52 pts with secondary FSGS, 8 pts with Alport-FSGS, 20 pts with obesity-related FSGS, 24 pts with diabetic nephropathy	Plasma and urinary suPAR levels in secondary FSGS group were significantly higher than in healthy controls - no significant difference between study groups - the plasma suPAR level was not correlated with eGFR and urine protein.	suPAR might be a useful marker of FSGS-associated podocytopathy but not necessarily a circulating permeability factor	[24]
	Plasma and urinary suPAR levels (ELISA)	241 pts with GN (Neptun cohort)	- plasma suPAR level at baseline inversely correlated with eGFR, - urine suPAR/creatinine ratio positively correlated with the urine protein/creatinine ratio	Results do not support a pathological role for suPAR in FSGS	[18]
	Serum suPAR level (ELISA)	476 non-FSGS CKD, 44 FSGS active disease, 10 FSGS remission	- eGFR is a potent determinant of suPAR levels	Results do not support a pathological role for suPAR in FSGS	[25]
	Serum suPAR level (ELISA)	69 Japanese pts with biopsy-proven glomerular diseases in a cross-sectional manner	- Lower suPAR levels in FSGS pts treated with steroids and/or immunosuppressants	suPAR do not differentiate primary FSGS from the other GN	[26]
	Plasma and urinary pretransplant suPAR levels (ELISA)	86 kidney transplant recipients, 10 healthy controls	- Serum and urine suPAR levels correlated with proteinuria and albuminuria - Serum suPAR elevated in all transplant candidates compared with healthy controls but not differentiate disease diagnosis - Urine suPAR was elevated in cases of recurrent FSGS compared with all other causes of ESRD.	Results do not support a pathological role for suPAR in FSGS	[27]
**CLC-1** **(experimental)**	Isolated rat glomeruli using an *in vitro* assay of albumin permeability (P(alb))	Rat model	The downregulation of JAK2/STAT3 signaling could relate to the circulating permeability factor by inhibiting CLC-1	Monomeric CLCF1 increases P(alb), the heterodimer CLCF1-CRLF1 may protect the glomerular filtration barrier	[28]

**Table 4:** Potential biomarkers in FSGS.

Biomarker	Pathophysiologic Mechanism	Clinical Utility	References
**Angiotensin II type 1 receptors (AT1R)**	- AT1R-Abs dysregulate AT1R receptors and lead to podocyte injury; in animals induce post-transplant FSGS; - The expression of AT1R in the kidney allograft biopsy correlated with a significantly higher risk of graft loss, serum levels of AT1R-Abs is increased in pts with recurrence FSGS after KTx; - Type D allele of the ACE gene is a risk factor for the progression to ESRD in patients with FSGS.	Prognostic factor of post-transplant FSGS prognostic factor of graft loss prognostic factor of in ESRD	[29,30,31,32,33,34,35]
**Metalloproteinases (MMP)/ tissue inhibitors of metalloproteinases (TIMPs)**	- Remodeling extracellular matrix and fibrosis; - Elevated expression was observed in podocytes during inflammation; - The level of MMPs and TIMPs were higher in patients with FSGS.	Differentiation FSGS with other glomerulopathies	[36,37,38,39,40,41]
**Dystroglycans** **(DG)**	- Alpha and beta DG anchors mechanically the podocyte cytoskeleton to the glomerular basement membrane; - Beta-DG transports important matrix proteins such as: perlecan, agrin, proteoglycans; - Increased DG expression in renal biopsies in patients with FSGS.	Differentiation FSGS with other glomerulopathies	[42,43,44,45]
**MicroRNAs (miR-192 and miR205, miR-186)**	- Take part in processes occurring after renal transplantation, including delayed graft function; - Overexpression of miR-193a induces foot process effacement in podocytes; - Serum miR-192 and miR205 levels were increased in FSGS pts versus MCD pts.	Prognostic factor of delayed graft function differentiation FSGS with other glomerulopathies	[46,47,48,49]
**Plasminogen activator inhibitor type-1 (PAI-1)**	- Deficiency or inhibition of PAI-1 activity causes remodeled plasmin generation and glomerulosclerosis; - PAI-1 mRNA expression correlated with the level of proteinuria in GN; - Glomerular PAI-1 mRNA expression was significantly higher in patients with MCD and FSGS; - Serum PAI-1 levels did not correlate with the clinical parameters in GN.	Potential role in the development of GN	[50,51,52,53,54]
**Forkheadbox P3 (FOXP3)**	- A transcription factor which influences the maintenance of the regulatory T-cell (Treg) phenotype and function; - Tregs and FOXP3 play a role in the pathogenesis of proliferative and crescentic forms of glomerulonephritis.	Identification of recipients at high risk for acute rejection; predictive marker kidney allograft outcome.	[55,56]
**Transforming growth factor-beta (TGF-β)**	Promotion of podocyte apoptosis, proliferation and matrix deposition; glomerular hypertrophy, extracellular matrix accumulation in tubulointerstitium and interstitial fibrosis	Predictive marker of renal failure in FSGS; predictive marker of response to steroid treatment	[57,58,59,60,61,62,63,64,65,66]
**Human neutrophil gelatinase-associated lipocalin (NGAL)**	- NGAL is accumulated in the human proximal tubules, early released from tubular epithelial cells, occurs following damage, levels of NGAL expression correlate with the degree of kidney injury	Prognostic factor of AKI and ESRD prognostic factor of graft loss biomarker of tubulointerstitial lesions in FSGS pts	[16,47,67,68,69,70,71,72,73,74,75]
**Malondialdehyde (MDA)**	- MDA is a biomarker of oxidative stress; causes lipid peroxidation; - Elevated levels of MDA in plasma, urine, and glomeruli in patients with FSGS with normal kidney function.	Prognostic factor of glomerulosclerosis in FSGS	[73,76]

## Data Availability

Not applicable.

## References

[1] Kim J.S., Han B.G., Choi S.O., Cha S.K. (2016). Secondary focal segmental glomerulosclerosis: From podocyte injury to glomerulosclerosis. Biomed. Res. Int..

[2] Beaudreuil S., Lorenzo H.K., Elias M., Obada E.N., Charpentier B., Durrbach A. (2017). Optimal management of primary focal segmental glomerulosclerosis in adults. Int. J. Nephrol. Renovasc. Dis..

[3] Fogo A.B. (2015). Causes and pathogenesis of focal segmental glomerulosclerosis. Nat. Rev. Nephrol..

[4] Perkowska-Ptasinska A., Bartczak A., Wagrowska-Danilewicz M., Halon A., Okon K., Wozniak A., Danilewicz M., Karkoszka H., Marszałek A., Kowalewska J. (2017). Clinicopathologic correlations of renal pathology in the adult population of Poland. Nephrol. Dial. Transplant..

[5] Schena F.P. (1997). Survey of the Italian Registry of Renal Biopsies. Frequency of the renal diseases for 7 consecutive years. Nephrol. Dial. Transplant..

[6] Rivera F., López-Gómez J.M., Pérez-García R. (2004). Clinicopathologic correlations of renal pathology in Spain. Kidney Int..

[7] Maixnerova D., Jancova E., Skibova J., Rysava R., Rychlik I., Viklicky O., Merta M., Kolsky A., Reiterova J., Neprasova M. (2015). Nationwide biopsy survey of renal diseases in the Czech Republic during the years 1994–2011. J. Nephrol..

[8] Schenk H., Müller-Deile J., Schmitt R., Bräsen J.H., Haller H., Schiffer M. (2017). Removal of focal segmental glomerulosclerosis (FSGS) factor suPAR using CytoSorb. J. Clin. Apher..

[9] Kdigo—Kidney Disease|Improving Global Outcomes. https://www.kidney-international.org/article/S0085-2538.

[10] Wen Y., Shah S., Campbell K.N., Campbell K.N. (2018). Molecular Mechanisms of Proteinuria in Focal Segmental Glomerulosclerosis. Front. Med..

[11] Wharram B.L., Goyal M., Wiggins J.E., Sanden S.K., Hussain S., Filipiak W.E., Saunders T.L., Dysko R.C., Kohno K., Holzman L.B. (2005). Podocyte Depletion Causes Glomerulosclerosis: Diphtheria Toxin—Induced Podocyte Depletion in Rats Expressing Human Diphtheria Toxin Receptor Transgene. J. Am. Soc. Nephrol..

[12] Kriz W. (2003). The pathogenesis of “classic” focal segmental glomerulosclerosis—Lessons from rat models. Nephrol. Dial. Transplant..

[13] De Vriese A.S., Sethi S., Nath K.A., Glassock R.J., Fervenza F.C. (2018). Differentiating primary, genetic, and secondary FSGS in adults: A clinicopathologic approach. J. Am. Soc. Nephrol..

[14] Shabaka A., Ribera A.T., Fernández-Juárez G. (2020). Focal Segmental Glomerulosclerosis: State-of-the-Art and Clinical Perspective. Nephron.

[15] Kitzler T.M., Kachurina N., Bitzan M.M., Torban E., Goodyer P.R. (2018). Use of genomic and functional analysis to characterize patients with steroid-resistant nephrotic syndrome. Pediatr. Nephrol..

[16] Wei C., Hindi S.E., Li J., Fornoni A., Goes N., Sageshima J., Maiguel D., Karumanchi S.A., Yap H., Saleem M. (2011). Circulating urokinase receptor as a cause of focal segmental glomerulosclerosis. Nat. Med..

[17] Harel E., Shoji J., Abraham V., Miller L., Laszik Z.G., King A., Dobi D., Szabo G., Hann B., Sarwal M.M. (2020). Further Evidence That the Soluble Urokinase Plasminogen Activator Receptor Does Not Directly Injure Mice or Human Podocytes. Transplantation.

[18] Spinale J.M., Mariani L.H., Kapoor S., Zhang J., Weyant R., Song P.X., Wong H.N., Troost J.P., Gadegbeku C.A., Gipson D.S. (2015). A reassessment of soluble urokinase-type plasminogen activator receptor in glomerular disease. Kidney Int..

[19] Harel E., Shoji J., Abraham V., Miller L., Laszik Z., Thurison T., King A., Olshen A., Leung J., Szabo G. (2019). Identifying a potential biomarker for primary focal segmental glomerulosclerosis and its association with recurrence after transplantation. Clin. Transplant..

[20] Huang J., Liu G., Zhang Y.M., Cui Z., Wang F., Liu X.J., Chu R., Chen Y., Zhao M.H. (2013). Plasma soluble urokinase receptor levels are increased but do not distinguish primary from secondary focal segmental glomerulosclerosis. Kidney Int..

[21] Wei C., Trachtman H., Li J., Dong C., Friedman A.L., Gassman J.J., Mcmahan J.L., Radeva M., Heil K.M., Trautmann A. (2012). Circulating suPAR in Two Cohorts of Primary FSGS. JASN.

[22] Hladunewich M.A., Cattran D., Sethi S.M., Hayek S.S., Li J., Wei C., Mullin S.I., Reich H.N., Reiser J., Fervenza F.C. (2021). Efficacy of Rituximab in Treatment-Resistant Focal Segmental Glomerulosclerosis with Elevated Soluble Urokinase-Type Plasminogen Activator Receptor and Activation of Podocyte β3 Integrin. Kidney Int. Rep..

[23] Winnicki W., Sunder-Plassmann G., Sengölge G., Handisurya A., Herkner H., Kornauth C., Bielesz B., Wagner L., Kikić Ž., Pajenda S. (2019). Diagnostic and Prognostic Value of Soluble Urokinase-type Plasminogen Activator Receptor (suPAR) in Focal Segmental Glomerulosclerosis and Impact of Detection Method. Sci. Rep..

[24] Sun P., Yu L., Huang J., Wang S., Zou W., Yang L., Liu G. (2019). Soluble Urokinase Receptor Levels in Secondary Focal Segmental Glomerulosclerosis. Kidney Dis..

[25] Meijers B., Maas R.J.H., Sprangers B., Claes K., Poesen R., Bammens B., Naesens M., Deegens J.K.J., Dietrich R., Storr M. (2014). The soluble urokinase receptor is not a clinical marker for focal segmental glomerulosclerosis. Kidney Int..

[26] Wada T., Nangaku M., Maruyama S., Imai E., Shoji K., Kato S., Endo T., Muso E., Kamata K., Yokoyama H. (2014). A multicenter cross-sectional study of circulating soluble urokinase receptor in Japanese patients with glomerular disease. Kidney Int..

[27] Franco Palacios C.R., Lieske J.C., Wadei H.M., Rule A.D., Fervenza F.C., Voskoboev N., Garovic V.D., Zand L., Stegall M.D., Cosio F.G. (2013). Urine but not serum soluble urokinase receptor (suPAR) may identify cases of recurrent FSGS in kidney transplant candidates. Transplantation.

[28] Sharma M., Zhou J., Gauchat J.F., Sharma R., McCarthy E.T., Srivastava T., Savin V.J. (2015). Janus kinase 2/signal transducer and activator of transcription 3 inhibitors attenuate the effect of cardiotrophin-like cytokine factor 1 and human focal segmental glomerulosclerosis serum on glomerular filtration barrier. Transl. Res..

[29] Nijenhuis T., Sloan A.J., Hoenderop J.G.J., Flesche J., Van Goor H., Kistler A.D., Bakker M., Bindels R.J.M., De Boer R.A., Möller C.C. (2011). Angiotensin II contributes to podocyte injury by increasing TRPC6 expression via an NFAT-mediated positive feedback signaling pathway. Am. J. Pathol..

[30] Zhou C.C. (2012). Eclampsia in Pregnant Mice. Nat. Med..

[31] Sas A., Donizy P., Kościelska-Kasprzak K., Kamińska D., Mazanowska O., Krajewska M., Chudoba P., Korta K., Hałoń A., Klinger M. (2018). Histopathological Relevance of Angiotensin II Type 1 Receptor in Renal Transplant Biopsy. Transplant. Proc..

[32] Sas-Strózik A., Donizy P., Kościelska-Kasprzak K., Kamińska D., Gawlik K., Mazanowska O., Madziarska K., Hałoń A., Krajewska M., Banasik M. (2020). Angiotensin II Type 1 Receptor Expression in Renal Transplant Biopsies and Anti-AT1R Antibodies in Serum Indicates the Risk of Transplant Loss. Transplant. Proc..

[33] Mujtaba M.A., Sharfuddin A.A., Book B.L., Goggins W.C., Khalil A.A., Mishler D.P., Fridell J.A., Yaqub M.S., Taber T.E. (2015). Pre-transplant angiotensin receptor II type 1 antibodies and risk of post-transplant focal segmental glomerulosclerosis recurrence. Clin. Transplant..

[34] Abuzeineh M., Aala A., Alasfar S., Alachkar N. (2020). Angiotensin II receptor 1 antibodies associate with post-transplant focal segmental glomerulosclerosis and proteinuria. BMC Nephrol..

[35] Frishberg Y., Becker-cohen R., Halle D., Feigin E., Eisenstein B., Halevy R., Lotan D., Juabeh I., Ish-shalom N., Magen D. (1998). Genetic polymorphisms of the renin-angiotensin system and the outcome of focal segmental glomerulosclerosis in children. Kidney Int..

[36] Keeling J., Herrera G.A. (2008). Human matrix metalloproteinases: Characteristics and pathologic role in altering mesangial homeostasis. Microsc. Res. Tech..

[37] Tallant C., Marrero A., Gomis-Rüth F.X. (2010). Matrix metalloproteinases: Fold and function of their catalytic domains. Biochim. Biophys. Acta Mol. Cell Res..

[38] Bauvois B., Mothu N., Nguyen J., Nguyen-Khoa T., Nöel L.H., Jungers P. (2007). Specific changes in plasma concentrations of matrix metalloproteinase-2 and -9, TIMP-1 and TGF-β1 in patients with distinct types of primary glomerulonephritis. Nephrol. Dial. Transplant..

[39] Liu S., Li Y., Zhao H., Chen D., Huang Q., Wang S., Zou W., Zhang Y., Li X., Huang H. (2006). Increase in extracellular cross-linking by tissue transglutaminase and reduction in expression of MMP-9 contribute differentially to focal segmental glomerulosclerosis in rats. Mol. Cell. Biochem..

[40] Chang H.R., Kuo W.H., Hsieh Y.S., Yang S.F., Lin C.C., Lee M.L., Lian J.D., Chu S.C. (2008). Circulating matrix metalloproteinase-2 is associated with cystatin C level, posttransplant duration, and diabetes mellitus in kidney transplant recipients. Transl. Res..

[41] Czech K.A., Bennett M., Devarajan P. (2011). Distinct metalloproteinase excretion patterns in focal segmental glomerulosclerosis. Pediatr. Nephrol..

[42] Gee S.H., Montanaro F., Lindenbaum M.H., Carbonetto S. (1994). Dystroglycan-α, a dystrophin-associated glycoprotein, is a functional agrin receptor. Cell.

[43] Timpl R., Tisi D., Talts J.F., Andac Z., Sasaki T., Hohenester E. (2000). Structure and function of laminin LG modules. Matrix Biol..

[44] Regele H.M., Fillipovic E., Langer B., Poczewki H., Kraxberger I., Bittner R.E., Kerjaschki D. (2000). Glomerular expression of dystroglycans is reduced in minimal change nephrosis but not in focal segmental glomerulosclerosis. J. Am. Soc. Nephrol..

[45] Shankar P.B., Nada R., Joshi K., Kumar A., Rayat C.S., Sakhuja V. (2014). Podocin and Beta Dystroglycan expression to study Podocyte-Podocyte and basement membrane matrix connections in adult protienuric states. Diagn. Pathol..

[46] Wilflingseder J., Reindl-schwaighofer R., Sunzenauer J. (2015). Europe PMC Funders Group microRNAs in Kidney Transplantation. Nephrol. Dial. Transplant..

[47] Zhang Q., Jiang C., Tang T., Wang H., Xia Y., Shao Q., Zhang M. (2017). Author’ s Accepted Manuscript. Am. J. Med. Sci..

[48] Gebeshuber C.A., Kornauth C., Dong L., Sierig R., Seibler J., Reiss M., Tauber S., Bilban M., Wang S., Kain R. (2013). Focal segmental glomerulosclerosis is induced by microRNA-193a and its downregulation of WT1. Nat. Med..

[49] Iranzad R., Motavalli R., Ghassabi A., Pourakbari R., Etemadi J., Yousefi M. (2021). Roles of microRNAs in renal disorders related to primary podocyte dysfunction. Life Sci..

[50] Flevaris P., Vaughan D. (2017). The Role of Plasminogen Activator Inhibitor Type-1 in Fibrosis. Semin. Thromb. Hemost..

[51] Hamano K., Iwano M., Akai Y., Sato H., Kubo A., Nishitani Y., Uyama H., Yoshida Y., Miyazaki M., Shiiki H. (2002). Expression of glomerular plasminogen activator inhibitor type 1 in glomerulonephritis. Am. J. Kidney Dis..

[52] Lee J.H., Oh M.H., Park J.S., Na G.J., Gil H.W., Yang J.O., Lee E.Y., Hong S.Y. (2014). Urokinase, urokinase receptor, and plasminogen activator inhibitor-1 expression on podocytes in immunoglobulin A glomerulonephritis. Korean J. Intern. Med..

[53] Pan C., Zhou C., Shan A., Chen N. (2020). Expression and clinical significance of thrombospondin-1 and plasminogen activator inhibitor-1 in patients with mesangial proliferative glomerulonephritis. Ann. Cardiothorac. Surg..

[54] Huang Y., Haraguchi M., Lawrence D.A., Border W.A., Yu L., Noble N.A. (2003). A mutant, noninhibitory plasminogen activator inhibitor type 1 decreases matrix accumulation in experimental glomerulonephritis. J. Clin. Investig..

[55] Paust H.J., Ostmann A., Erhardt A., Turner J.E., Velden J., Mittrücker H.W., Sparwasser T., Panzer U., Tiegs G. (2011). Regulatory T cells control the Th1 immune response in murine crescentic glomerulonephritis. Kidney Int..

[56] Barabadi M., Shahbaz S.K., Foroughi F., Hosseinzadeh M., Nafar M., Yekaninejad M.S., Amirzargar A. (2018). High expression of FOXP3 mRNA in blood and urine as a predictive marker in kidney transplantation. Prog. Transplant..

[57] Koch A., Voigt S., Kruschinski C., Sanson E., Dückers H., Horn A., Yagmur E., Zimmermann H., Trautwein C., Tacke F. (2011). Circulating soluble urokinase plasminogen activator receptor is stably elevated during the first week of treatment in the intensive care unit and predicts mortality in critically ill patients. Crit. Care.

[58] Yin J., Tu J., Lin H., Shi F., Liu R., Zhao C., Stephen W., Kuniyoshi S., Shi J. (2010). Centrally Administered Pertussis Toxin Inhibits Microglia Migration to the Spinal Cord and Prevents Dissemination of Disease in an EAE Mouse Model. PLoS ONE.

[59] Becker L.E., Koleganova N., Piecha G., Noronha I.L., Zeier M., Geldyyev A., Kökeny G., Ritz E., Gross M.L. (2011). Effect of paricalcitol and calcitriol on aortic wall remodeling in uninephrectomized ApoE knockout mice. Am. J. Physiol. Ren. Physiol..

[60] Nakamaki S., Satoh H., Kudoh A., Hayashi Y., Hirai H., Watanabe T. (2011). Adiponectin reduces proteinuria in streptozotocin-induced diabetic wistar rats. Exp. Biol. Med..

[61] Marwitz S., Abdullah M., Vock C., Fine J.S., Visvanathan S., Gaede K.I., Hauber H.P., Zabel P., Goldmann T. (2011). HOPE-BAL: Improved molecular diagnostics by application of a novel technique for fixation and paraffin embedding. J. Histochem. Cytochem..

[62] Vieira V.J., D’Acampora A.J., Marcos A.B.W., Di Giunta G., De Vasconcellos Z.A.A., Bins-Ely J., D’Eça Neves R., Figueiredo C.P. (2010). Vascular endothelial growth factor overexpression positively modulates the characteristics of periprosthetic tissue of polyurethane-coated silicone breast implant in rats. Plast. Reconstr. Surg..

[63] Yamamoto T., Noble N.A., Cohen A.H., Nast C.C., Hishida A.M.I., Gold L.I., Border W.A. (1996). Expression of transforming growth factor-n isoforms in human glomerular diseases. Kidney Int..

[64] Wu D.T., Bitzer M., Ju W., Mundel P., Bo E.P. (2005). Profiles of Growth Arrest/Differentiation and Apoptosis in Podocytes. JASN.

[65] Mozes M.M., Böttinger E.P., Jacot T.A., Kopp J.B. (1999). Renal expression of fibrotic matrix proteins and of transforming growth factor-β (TGF-β) isoforms in TGF-β transgenic mice. J. Am. Soc. Nephrol..

[66] Strehlau J., Schachter A.D., Pavlakis M., Singh A., Tejani A., Strom T.B. (2002). Activated intrarenal transcription of CTL-effectors and TGF-ß1 in children with focal segmental glomerulosclerosis. Kidney Int..

[67] Donizy P., Wu C.L., Mull J., Fujimoto M., Chłopik A., Peng Y., Shalin S.C., Selim M.A., Puig S., Fernandez-Figueras M.T. (2020). Up-Regulation of PARP1 Expression Significantly Correlated with Poor Survival in Mucosal Melanomas. Cells.

[68] Flower D.R. (1996). The lipocalin protein family: Structure and function. Biochem. J..

[69] Kulvichit W., Kellum J.A., Srisawat N. (2021). Biomarkers in Acute Kidney Injury. Crit. Care Clin..

[70] Bennett M., Dent C.L., Ma Q., Dastrala S., Grenier F., Workman R., Syed H., Ali S., Barasch J., Devarajan P. (2008). Urine NGAL predicts severity of acute kidney injury after cardiac surgery: A prospective study. Clin. J. Am. Soc. Nephrol..

[71] Tryggvason K., Pettersson E. (2003). Causes and consequences of proteinuria: The kidney filtration barrier and progressive renal failure. J. Intern. Med..

[72] Ece A., Kelekçi S., Kocamaz H., Hekimoǧlu A., Balik H., Yolbaş I., Erel O. (2008). Antioxidant enzyme activities, lipid peroxidation, and total antioxidant status in children with Henoch-Schönlein purpura. Clin. Rheumatol..

[73] Wager MGT and JFS (2008). 基因的改变NIH Public Access. Bone.

[74] Chen B.Y., Lin D.P.C., Wu C.Y., Teng M.C., Sun C.Y., Tsai Y.T., Su K.C., Wang S.R., Chang H.H. (2011). Dietary zerumbone prevents mouse cornea from UVB-induced photokeratitis through inhibition of NF-κB, iNOS, and TNF-α expression and reduction of MDA accumulation. Mol. Vis..

[75] DeNicola G.M., Karreth F.A., Humpton T.J., Gopinathan A., Wei C., Frese K., Mangal D., Yu K.H., Yeo C.J., Eric S. (2012). HHS Public Access Author manuscript Nature. Nature.

[76] Kuo H.T., Kuo M.C., Chiu Y.W., Chang J.M., Guh J.Y., Chen H.C. (2005). Increased glomerular and extracellular malondialdehyde levels in patients and rats with focal segmental glomerulosclerosis. Eur. J. Clin. Investig..

[77] Da Silva C.A., Araújo L.S., Dos Reis Monteiro M.L.G., De Morais Pereira L.H., Da Silva M.V., Castellano L.R.C., Corrêa R.R.M., Dos Reis M.A., Machado J.R. (2019). Evaluation of the diagnostic potential of UPAR as a biomarker in renal biopsies of patients with FSGS. Dis. Markers.

[78] Wei C., Möller C.C., Altintas M.M., Li J., Schwarz K., Zacchigna S., Xie L., Henger A., Schmid H., Rastaldi M.P. (2008). Modification of kidney barrier function by the urokinase receptor. Nat. Med..

[79] Kong X., Zhang Y., Wu H.B., Li F.X., Zhang D.Y., Su Q. (2012). Combination therapy with losartan and pioglitazone additively reduces renal oxidative and nitrative stress induced by chronic high fat, sucrose, and sodium intake. Oxid. Med. Cell. Longev..

[80] McCarthy E.T., Sharma M., Savin V.J. (2010). Circulating permeability factors in idiopathic nephrotic syndrome and focal segmental glomerulosclerosis. Clin. J. Am. Soc. Nephrol..

[81] Elson G.C., Graber P., Losberger C., Herren S., Gretener D., Menoud L.N., Wells T.N., Kosco-Vilbois M.H., Gauchat J.F. (1998). Cytokine-like factor-1, a novel soluble protein, shares homology with members of the cytokine type I receptor family. J. Immunol..

[82] Bazan J.F. (1991). Neuropoietic cytokines in the hematopoietic fold. Neuron.

[83] Derouet D., Rousseau F., Alfonsi F., Froger J., Hermann J., Barbier F., Perret D., Diveu C., Guillet C., Preisser L. (2004). Neuropoietin, a new IL-6-related cytokine signaling through the ciliary neurotrophic factor receptor. Proc. Natl. Acad. Sci. USA.

[84] Stefanovic L., Stefanovic B. (2012). Role of cytokine receptor-like factor 1 in hepatic stellate cells and fibrosis. World J. Hepatol..

[85] Savin V.J., McCarthy E.T., Sharma R., Charba D., Sharma M. (2008). Galactose binds to focal segmental glomerulosclerosis permeability factor and inhibits its activity. Transl. Res..

[86] Sgambat K., Banks M., Moudgil A. (2013). Effect of galactose on glomerular permeability and proteinuria in steroid-resistant nephrotic syndrome. Pediatr. Nephrol..

[87] Wada T., Nangaku M. (2015). A circulating permeability factor in focal segmental glomerulosclerosis: The hunt continues. Clin. Kidney J..

[88] Trachtman H., Vento S., Herreshoff E., Radeva M., Gassman J., Stein D.T., Savin V.J., Sharma M., Reiser J., Wei C. (2015). Efficacy of galactose and adalimumab in patients with resistant focal segmental glomerulosclerosis: Report of the font clinical trial group Clinical Research. BMC Nephrol..

[89] Suzuki K., Gi D.H., Miyauchi N., Hashimoto T., Nakatsue T., Fujioka Y., Koike H., Shimizu F., Kawachi H. (2007). Angiotensin II type 1 and type 2 receptors play opposite roles in regulating the barrier function of kidney glomerular capillary wall. Am. J. Pathol..

[90] Dragun D., Müller D.N., Bräsen J.H., Fritsche L., Nieminen-Kelhä M., Dechend R., Kintscher U., Rudolph B., Hoebeke J., Eckert D. (2005). Angiotensin II Type 1–Receptor Activating Antibodies in Renal-Allograft Rejection. N. Engl. J. Med..

[91] Haessler S., Granowitz E.V. (2013). Norovirus Gastroenteritis in Immunocompromised Patients. N. Engl. J. Med..

[92] Berdeli A., Serdaro E. (2005). Effects of Genetic Polymorphisms of the Renin-Angiotensin System in Children with Nephrotic Syndrome. J. Renin. Angiotensin. Aldosterone Syst ..

[93] Urushihara M., Kagami S., Kuhara T., Tamaki T., Kuroda Y. (2002). Glomerular distribution and gelatinolytic activity of matrix metalloproteinases in human glomerulonephritis. Nephrol. Dial. Transplant..

[94] Catania J.M., Chen G., Parrish A.R. (2007). Role of matrix metalloproteinases in renal pathophysiologies. Am. J. Physiol. Ren. Physiol..

[95] Ahuja T.S., Gopalani A., Davies P., Ahuja H. (2003). Matrix metalloproteinase-9 expression in renal biopsies of patients with HIV-associated nephropathy. Nephron. Clin. Pract..

[96] Korzeniecka-Kozerska A., Wasilewska A., Tenderenda E., Sulik A., Cybulski K. (2013). Urinary MMP-9/NGAL ratio as a potential marker of FSGS in nephrotic children. Dis. Markers.

[97] (2008). Eton & Lepore 基因的改变NIH Public Access. Bone.

[98] Mazanowska O., Zabińska M., Kościelska-Kasprzak K., Kamińska D., Krajewska M., Banasik M., Madziarska K., Zmonarski S.C., Chudoba P., Biecek P. (2014). Increased plasma matrix metalloproteinase-2 (MMP-2), tissue inhibitor of proteinase-1 (TIMP-1), TIMP-2, and urine MMP-2 concentrations correlate with proteinuria in renal transplant recipients. Transplant. Proc..

[99] Tong J., Jin Y., Weng Q., Yu S., Jafar Hussain H.M., Ren H., Xu J., Zhang W., Li X., Wang W. (2020). Glomerular Transcriptome Profiles in Focal Glomerulosclerosis: New Genes and Pathways for Steroid Resistance. Am. J. Nephrol..

[100] Raats C.J.I., van den Born J., Bakker M.A.H., Oppers-Walgreen B., Pisa B.J.M., Dijkman H.B.P.M., Assmann K.J.M., Berden J.H.M. (2000). Expression of agrin, dystroglycan, and utrophin in normal renal tissue and in experimental glomerulopathies. Am. J. Pathol..

[101] Shimojima M., Stroher U., Ebihara H., Feldmann H., Kawaoka Y. (2012). Identification of Cell Surface Molecules Involved in Dystroglycan-Independent Lassa Virus Cell Entry. J. Virol..

[102] Takawira D., Budinger G.R.S., Hopkinson S.B., Jones J.C.R. (2011). A dystroglycan/plectin scaffold mediates mechanical pathway bifurcation in lung epithelial cells. J. Biol. Chem..

[103] Zanoteli E., van de Vlekkert D., Bonten E.J., Hu H., Mann L., Gomero E.M., Harris A.J., Ghersi G., d—Azzo A. (2011). Muscle degeneration in neuraminidase 1-deficient mice results from infiltration of the muscle fibers by expanded connective tissue. Biochim. Et Biophys. Acta (BBA) Mol. Basis Dis..

[104] Khouzami L., Bourin M.C., Christov C., Damy T., Escoubet B., Caramelle P., Perier M., Wahbi K., Meune C., Pavoine C. (2010). Delayed cardiomyopathy in dystrophin deficient mdx mice relies on intrinsic glutathione resource. Am. J. Pathol..

[105] Ribeiro-Resende V.T., Ribeiro-Guimarães M.L., Rodrigues Lemes R.M., Nascimento Í.C., Alves L., Mendez-Otero R., Vidal Pessolani M.C., Lara F.A. (2010). Involvement of 9-O-acetyl GD3 ganglioside in *Mycobacterium leprae* infection of schwann cells. J. Biol. Chem..

[106] Ilsley J.L., Sudol M., Winder S.J. (2002). The WW domain: Linking cell signalling to the membrane cytoskeleton. Cell. Signal..

[107] Zhang Z., Zhang P., Hu H. (2011). LARGE Expression Augments the Glycosylation of Glycoproteins in Addition to a -Dystroglycan Conferring Laminin Binding. PLoS ONE.

[108] Kojima K., Kerjaschki D. (2002). Is podocyte shape controlled by the dystroglycan complex?. Nephrol. Dial. Transplant..

[109] Giannico G., Yang H., Neilson E.G., Fogo A.B. (2009). Dystroglycan in the diagnosis of FSGS. Clin. J. Am. Soc. Nephrol..

[110] Cortez M.A., Calin G.A. (2009). MicroRNA identification in plasma and serum: A new tool to diagnose and monitor diseases. Expert Opin. Biol. Ther..

[111] Mitchell P.S., Parkin R.K., Kroh E.M., Fritz B.R., Wyman S.K., Pogosova-Agadjanyan E.L., Peterson A., Noteboom J., O’Briant K.C., Allen A. (2008). Circulating microRNAs as stable blood-based markers for cancer detection. Proc. Natl. Acad. Sci. USA.

[112] Nalewajska M., Gurazda K., Styczyńska-Kowalska E., Marchelek-Myśliwiec M., Pawlik A., Dziedziejko V. (2019). The role of microRNAs in selected forms of glomerulonephritis. Int. J. Mol. Sci..

[113] Li S., Wei X., He J., Tian X., Yuan S., Sun L. (2018). Plasminogen activator inhibitor-1 in cancer research. Biomed. Pharmacother..

[114] Lu L., Barbi J., Pan F. (2017). The regulation of immune tolerance by FOXP3. Nat. Rev. Immunol..

[115] Bunnag S., Allanach K., Jhangri G.S., Sis B., Einecke G., Mengel M., Mueller T.F., Halloran P.F. (2008). FOXP3 expression in human kidney transplant biopsies is associated with rejection and time post transplant but not with favorable outcomes. Am. J. Transplant..

[116] Iwase H., Kobayashi T., Kodera Y., Miwa Y., Kuzuya T., Iwasaki K., Haneda M., Katayama A., Takeda A., Morozumi K. (2011). Clinical significance of regulatory T-cell-related gene expression in peripheral blood after renal transplantation. Transplantation.

[117] Aparecida Da Silva C., Molinar Mauad Cintra M., De Castro Côbo E., Vinícius Da Silva M., Bichuette Custódio F., Rosa Miranda Corrêa R., Roberto Castellano L., Antônia Dos Reis M., Reis Machado J. (2014). Renal biopsy: Use of biomarkers as a tool for the diagnosis of focal segmental glomerulosclerosis. Dis. Markers.

[118] Donizy P., Halon A., Surowiak P., Pietrzyk G., Kozyra C., Matkowski R. (2013). Correlation between PARP-1 immunoreactivity and cytomorphological features of parthanatos, a specific cellular death in breast cancer cells. Eur. J. Histochem..

[119] Donizy P., Pietrzyk G., Halon A., Kozyra C., Gansukh T., Lage H., Surowiak P., Matkowski R. (2014). Nuclear-cytoplasmic PARP-1 expression as an unfavorable prognostic marker in lymph node-negative early breast cancer: 15-year follow-up. Oncol. Rep..

[120] Mukhopadhyay P., Horváth B., Kechrid M., Tanchian G., Rajesh M., Naura A.S., Boulares A.H., Pacher P. (2011). Poly(ADP-ribose) polymerase-1 is a key mediator of cisplatin-induced kidney inflammation and injury. Free Radic. Biol. Med..

[121] Salech F., Ponce D.P., Paula-Lima A.C., SanMartin C.D., Behrens M.I. (2020). Nicotinamide, a Poly [ADP-Ribose] Polymerase 1 (PARP-1) Inhibitor, as an Adjunctive Therapy for the Treatment of Alzheimer’s Disease. Front. Aging Neurosci..

[122] Pacher P., Szabó C. (2007). Role of poly(ADP-ribose) polymerase 1 (PARP-1) in cardiovascular diseases: The therapeutic potential of PARP inhibitors. Cardiovasc. Drug Rev..

[123] O’Valle F., Del Moral R.G.M., del Carmén Benítez M., Martín-Oliva D., Gómez-Morales M., Aguilar D., Aneiros-Fernández J., Hernández-Cortés P., Osuna A., Moreso F. (2009). Poly[ADP-ribose] polymerase-1 expression is related to cold ischemia, acute tubular necrosis, and delayed renal function in kidney transplantation. PLoS ONE.

[124] Pil S., Jinu Y. (2015). Poly (ADP-ribose) polymerase 1 activation links ischemic acute kidney injury to interstitial fibrosis. J. Physiol. Sci..

[125] Zheng J., Devalaraja-narashimha K., Singaravelu K., Padanilam B.J., Devalaraja-narashimha K., Sin- K., Adp-ribose B.J.P.P. (2005). Poly (ADP-ribose) polymerase-1 gene ablation protects mice from ischemic renal injury. Am. J. Physiol. -Ren. Physiol..

[126] Slominska E.M., Kowalik K., Smolenski R.T., Szolkiewiez M., Rutkowski P., Rutkowski B., Swierczynski J. (2006). Accumulation of poly(ADP-ribose) polymerase inhibitors in children with chronic renal failure. Pediatr. Nephrol..

[127] Li Y., Kang Y.S., Dai C., Kiss L.P., Wen X., Liu Y. (2008). Epithelial-to-mesenchymal transition is a potential pathway leading to podocyte dysfunction and proteinuria. Am. J. Pathol..

[128] Kamińska D., Kościelska-Kasprzak K., Chudoba P., Mazanowska O., Banasik M., Zabinska M., Boratyńska M., Lepiesza A., Gomółkiewicz A., Dziȩgiel P. (2016). Pretransplant immune- and apoptosis-related gene expression is associated with kidney allograft function. Mediators Inflamm..

[129] Kamińska D., Kościelska-Kasprzak K., Chudoba P., Hałoń A., Mazanowska O., Gomółkiewicz A., Dzięgiel P., Drulis-Fajdasz D., Myszka M., Lepiesza A. (2016). The influence of warm ischemia elimination on kidney injury during transplantation—clinical and molecular study. Sci. Rep..

[130] Murakami K., Takemura T., Hino S., Yoshioka K. (1997). Urinary transforming growth factor-β in patients with glomerular diseases. Pediatr. Nephrol..

[131] Anderson S., Meyer T.W., Rennke H.G., Brenner B.M. (1985). Control of glomerular hypertension limits glomerular injury in rats with reduced renal mass. J. Clin. Investig..

[132] Remuzzi A., Puntorieri S., Battaglia C., Bertani T., Remuzzi G. (1990). Angiotensin converting enzyme inhibition ameliorates glomerular filtration of macromolecules and water and lessens glomerular injury in the rat. J. Clin. Investig..

[133] Le Berre L., Hervé C., Buzelen F., Usal C., Soulillou J.P., Dantal J. (2005). Renal macrophage activation and Th2 polarization precedes the development of nephrotic syndrome in Buffalo/Mna rats. Kidney Int..

[134] Kim J.H., Kim B.K., Moon K.C., Hong H.K., Lee H.S. (2003). Activation of the TGF-β/Smad signaling pathway in focal segmental glomerulosclerosis. Kidney Int..

[135] Wotton D., Massague J. (2000). Transcriptional control by the TGF- b / Smad signaling system. EMBO J..

[136] Schiffer M., Bitzer M., Roberts I.S.D., Kopp J.B., Dijke P., Mundel P., Böttinger E.P. (2001). Apoptosis in podocytes induced by TGF-β and Smad7. J. Clin. Investig..

[137] Khalili M., Bonnefoy A., Genest D.S., Quadri J., Rioux J.P., Troyanov S. (2020). Clinical Use of Complement, Inflammation, and Fibrosis Biomarkers in Autoimmune Glomerulonephritis. Kidney Int. Rep..

[138] Youssef D.M., El-shal A.S. (2012). Kidney Diseases Urinary Neutrophil Gelatinase-associated Lipocalin and Kidney Injury in Children With Focal Segmental Glomerulosclerosis. Iran. J. Kidney Dis..

[139] Neria F., Castilla M.A., Sanchez R.F., Gonzalez Pacheco F.R., Deudero J.J.P., Calabia O., Tejedor A., Manzarbeitia F., Ortiz A., Caramelo C. (2009). Inhibition of JAK2 protects renal endothelial and epithelial cells from oxidative stress and cyclosporin A toxicity. Kidney Int..

[140] Mancuso M., Davidzon G., Kurlan R.M., Tawil R., Bonilla E., Di Mauro S., Powers J.M. (2005). Hereditary ferritinopathy: A novel mutation, its cellular pathology, and pathogenetic insights. J. Neuropathol. Exp. Neurol..

